# Spatial Patterns of Satellite-Retrieved PM_2.5_ and Long-Term Exposure Assessment of China from 1998 to 2016

**DOI:** 10.3390/ijerph15122785

**Published:** 2018-12-08

**Authors:** Tan Chen, Shulin Deng, Manchun Li

**Affiliations:** 1 Jiangsu Provincial Key Laboratory of Geographic Information Science and Technology, Nanjing University, Nanjing 210023, China; tanchen@smail.nju.edu.cn (T.C.); dengshulin12531@163.com (S.D.); 2 School of Geography and Ocean Science, Nanjing University, Nanjing 210023, China; 3 Collaborative Innovation Center of South China Sea Studies, Nanjing University, Nanjing 210093, China

**Keywords:** PM_2.5_, standard deviational ellipse analysis, health impact assessment, benefits analysis

## Abstract

Previous studies have shown that particulate matter with an aerodynamic diameter of less than 2.5 micrometers (PM_2.5_) is tightly associated with adverse effects on human health, i.e., morbidity and mortality. Based on long-term satellite-derived PM_2.5_ datasets, this study analyzed the spatial patterns and temporal trends of PM_2.5_ concentrations in China from 1998 to 2016 using standard deviational ellipse and statistical analyses. A long-term assessment of exposure and health impacts due to PM_2.5_ was undertaken by the Environmental Benefits Mapping and Analysis Program-Community Edition (BenMAP-CE) model. The results show that concentrations of PM_2.5_ increased nonlinearly in most areas of China from 1998 to 2016. Higher concentrations were found in eastern China and western Tarim Basin, and most areas exceeded the World Health Organization’s (WHO) annual PM_2.5_ standards. The median center of average PM_2.5_ concentration of the country shifted to the southeast and then returned during the examined time period. The proportion of the population exposed to equal PM_2.5_ concentrations increased at first, then trended downward. The proportion of the population exposed to PM_2.5_ over WHO Interim Target-1 (35 µg/m^3^) increased 20.6%, which was the largest growth compared with other WHO standard levels. The extent of health risk in China increased and expanded from 1998 to 2016, especially in the Beijing-Tianjin-Hebei region, the Yangtze River Delta, and the Pearl River Delta, which are China’s top three urban areas. The implementation of the Air Pollution Prevention and Control Action Plan has gradually paid off. If the government can achieve long-term adherence to its plan, great economic and health benefits will be gotten through the BenMAP-CE model analysis.

## 1. Introduction

The relationship between air pollution and human health is a topic of lively debate among policy makers and researchers. Long-term exposure to particulate matter with an aerodynamic diameter of less than 2.5 micrometers (PM_2.5_) can affect human health [1,2,3]. The World Health Organization (WHO) estimates that around 4.2 million people died in 2016 due to outdoor air pollution [4]. PM_2.5_ penetrates deep into the lungs and cardiovascular system, causing diseases such as stroke, heart disease, lung cancer, chronic obstructive pulmonary disease, and respiratory infections, including pneumonia [4]. Under the WHO Global Burden of Disease (GBD) project, air pollution is considered a high-priority area and PM_2.5_ is considered to be one of the leading risk factors for premature mortality [5]. The GBD estimates that PM_2.5_ was responsible for 4.24 million deaths and 103.1 million disability-adjusted life years globally in 2015 [6]. Therefore, understanding the spatial patterns and long-term trends of PM_2.5_ concentrations is essential for risk assessment.

Mapping (by, e.g., a local regression, interpolation approach) and remote sensing can overcome the issue of heterogeneous spatial distribution of monitoring stations to evaluate/quantify the population’s exposure to PM [7,8,9]. With satellite launches and continuous improvements in data retrieval technologies, remote sensing of PM_2.5_ can supplement traditional observations. The remote sensing inversion method has the advantage of quickly obtaining broad spatial coverage data compared with ground-based monitoring. Scientists have developed methods for inferring ground-level PM_2.5_ concentrations from satellite-derived aerosol optical depth (AOD) measurements [10,11]. Currently, AOD retrieval products from Moderate-Resolution Imaging Spectroradiometer (MODIS), Multi-angle Imaging Spectroradiometer (MISR), and Sea-Viewing Wide Field-of-View Sensor (SeaWiFS) instruments are widely used for inversion of PM_2.5_ [12]. Van Donkelaar et al. combined multivariate AOD products to estimate global concentrations of PM_2.5_ [13].

Previous studies have characterized spatiotemporal patterns of PM_2.5_ on different temporal and spatial scales. On a regional scale, Yang et al. characterized PM_2.5_ in southern Jiangsu Province in eastern China, and Du et al. investigated the direct and spillover effect of urbanization on PM_2.5_ concentrations in three urban agglomerations of China [14,15]. On a national scale, Peng et al. reported spatiotemporal patterns of PM_2.5_ over China from 1999 to 2011 [16]. On a continent scale, Shi et al. found rising levels of PM_2.5_ in South and Southeast Asia between 1999 and 2014 [17]. On a global scale, van Donkelaar et al. demonstrated that satellite observations provide insight into global long-term changes in ambient PM_2.5_ concentrations [13]. However, studies related to PM_2.5_ on a large scale and at finer spatial resolution over longer time periods are still lacking. New publicly available global remote sensing data with high resolution (0.01° × 0.01° approach to 1 km) and a long-term period (1998–2016) developed by van Donkelaar et al. provides an opportunity for the research conducted here [18].

Numerous studies have estimated the impacts of ambient PM_2.5_ on human health worldwide [19,20,21,22,23]. Previous GBD estimates adopted log models, which were proposed by Cohan et al. and are currently recommended by the WHO [24,25]. Daryanoosh et al. (2017) calculated morbidity attributed to ambient PM_10_ in Iran using the AirQ model, implemented by WHO and based on incidence and relative risk values for given health endpoints [26]. Burnett et al. (2014) fitted an integrated exposure–response model by integrating available relative risk information from studies of ambient air pollution [27]. However, previous estimates of mortality attributed to particulate matter based on exposure reaction functions required that researchers had a high level of experience in epidemiology. In several studies, health impacts and their monetary value were estimated by the US Environmental Protection Agency’s (EPA) health and environmental assessment model, known as the Environmental Benefits Mapping and Analysis Program (BenMAP) [28,29,30]. BenMAP provides flexibility to perform a broad array of analyses at the local, regional, national, and global scale. It is mostly used by international researchers to assess the human health impacts and conduct benefits analyses of air pollution [31,32,33]. In addition, based on Geographic Information System (GIS) assessment, BenMAP tools can easily obtain continuous surface values compared with single-point health evaluations. Most studies assessing human health impact due to PM_2.5_ were conducted with coarse resolution based on ground station data. The simulation results of an air quality model can improve imaging spatial resolution of BenMAP assessment outputs relative to observations. However, it is limited by uncertainties of a high-resolution and large-scale emissions inventory. Therefore, in this study we apply high-spatial-resolution satellite-retrieved PM_2.5_ instead of simulation data and ground-based data to the BenMAP model to improve health assessment results.

This study investigates the spatiotemporal patterns and variations of PM_2.5_ concentrations in China with a high spatial resolution of 0.01° × 0.01° and estimates the associated health impacts. The specific research objectives were to (1) reveal the dynamic characteristics of PM_2.5_ concentrations in China from 1998 to 2016 by standard deviational ellipse (SDE) analysis, (2) examine the proportion of the population exposed to PM_2.5_ levels exceeding WHO standards, and (3) calculate health and monetary impacts based on BenMAP.

## 2. Materials and Methods

### 2.1. Study Area

**Figure 1:**
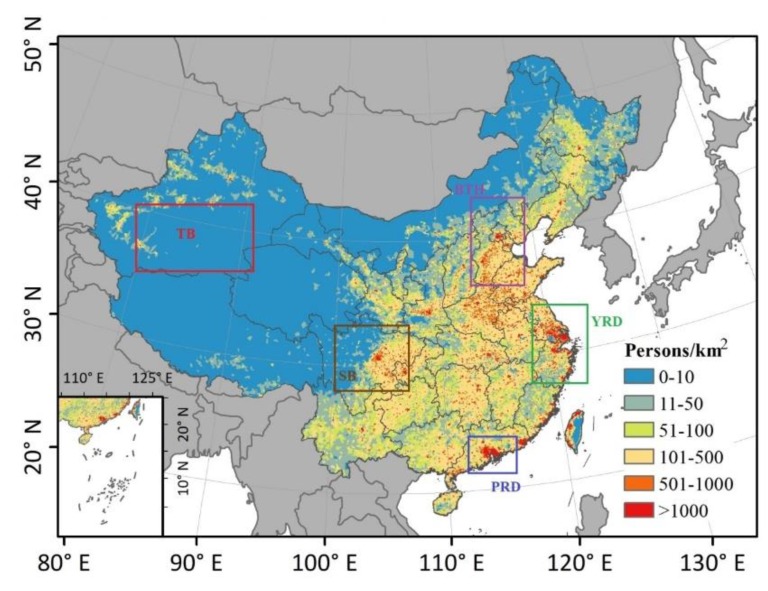
Study area with population distribution averaged from 1998 to 2016 at 1 km resolution. TB, Tarim Basin; SB, Sichuan Basin; BTH, Beijing-Tianjin-Hebei region; YRD, Yangtze River Delta; PRD, Pearl River Delta.

China is located in Southeast Asia along the coastline of the Pacific Ocean with an area of 9.6 million square kilometers and a coastline of 18,000 kilometers. It has the largest population in the world today and approximately 1.4 billion people. The Tarim Basin is an endorheic basin in the northwest of China which is dominated by Taklamakan Desert. The top three urban agglomerations Beijing-Tianjin-Hebei (BTH), Yangtze River Delta (YRD), and Pearl River Delta (PRD) are situated in eastern coastal areas of China (Figure 1). BTH, YRD, PRD and densely populated Sichuan Basin occupy the majority of the urban population of China. 

### 2.2. Data

#### 2.2.1. Satellite-Retrieved PM_2.5_

Annual mean satellite-retrieved PM_2.5_ concentrations with a spatial resolution of 1 km from 1998 to 2016 are freely available from the Dalhousie University Atmospheric Composition Analysis Group website (http://fizz.phys.dal.ca/~atmos/martin/?page_id=140). The PM_2.5_ concentrations were estimated by combining aerosol optical depth (AOD) retrievals from the NASA MODIS, MISR, and SeaWiFS instruments with the GEOS-Chem chemical transport model and subsequently calibrated to global ground-based observations of PM_2.5_ using geographically weighted regression (GWR) [18]. The PM_2.5_ remote-sensing dataset was inversed by van Donkelaar et al. with the largest coverage and longest time span available, which has been validated and can be effectively applied on a national scale [13].

#### 2.2.2. Population Data

Gridded population count estimates at 1 × 1 km resolution were derived from the Socioeconomic Data and Applications Center (SEDAC; http://sedac.ciesin.columbia.edu/data/collection/gpw-v4). The Gridded Population of the World collection, now in its fourth version (GPWv4), models the distribution of human population (counts and densities) on a continuous global raster surface. GPWv4 is gridded with an output resolution of 30 arc-seconds (approximately 1 km at the equator) for the years 2000, 2005, 2010, 2015, and 2020. We estimated and resampled the population data onto a 1 km grid for other study years using linear interpolation.

### 2.3. Methods

#### 2.3.1. Standard Deviational Ellipse Analysis

The standard deviational ellipse (SDE) was developed by Lefever (1926) to analyze distribution characteristics of discrete point data [34]. With the development of its application, the SDE method has long served as a versatile GIS tool for delineating the geographic distribution of concerned features [35]. When drawing the features on a map, calculating the standard deviational ellipse makes the directional trend clear. Specifically, the SDE features include median center, major axis, minor axis, and azimuth. Among these features, the median center is the center of spatial data, which indicates the gravity of the distribution; the major and minor axes of the ellipses indicate the directions and ranges of the data distribution; and the azimuth reflects the main trend directions [16,17]. Thus, the SDE method was used to trace the changes in spatial patterns of PM_2.5_ concentrations across a time series.

The standard deviational ellipse is given as:

(1) $${\mathrm{SDE}}_{x}=\sqrt{\frac{\sum_{i\;=\;1}^{n}{{(x}_{i\;}-\bar{\;x})}^{2}}{n}}$$

(2) $${\mathrm{SDE}}_{y}=\sqrt{\frac{\sum_{i\;=\;1}^{n}{\left(y_{i}\;-\bar{\;y}\right)}^{2}}{n}}$$

where *x_i_* and *y_i_* are the coordinates for feature *i*, {$\bar{x}$, $\bar{y}$} represents the median center for the features, and *n* is equal to the total number of features.

The angle of rotation is calculated as:

(3) $$\tan\theta =\frac{\left(\sum_{i=1}^{n}{\tilde{x}}_{i}^{2}-\sum_{i=1}^{n}{\tilde{y}}_{i}^{2}\right)+\sqrt{{\left(\sum_{i=1}^{n}{\tilde{x}}_{i}^{2}-\sum_{i=1}^{n}{\tilde{y}}_{i}^{2}\right)}^{2}+4{\left(\sum_{i=1}^{n}{\tilde{x}}_{i}{\tilde{y}}_{i}\right)}^{2}}}{2\sum_{i=1}^{n}{\tilde{x}}_{i}{\tilde{y}}_{i}}$$

where ${\tilde{x}}_{i}$ and ${\tilde{y}}_{i}$ are the deviations of the *x* and *y* coordinates from the median center.

The standard deviations for the *x*-axis and *y*-axis are:

(4) $$\sigma_{x}=\sqrt{\frac{\sum_{i=1}^{n}{\left({\tilde{x}}_{i}\cos\theta -{\tilde{y}}_{i}\sin\theta \right)}^{2}}{n}}$$

(5) $$\sigma_{y}=\sqrt{\frac{\sum_{i=1}^{n}{\left({\tilde{x}}_{i}\sin\theta -{\tilde{y}}_{i}\cos\theta \right)}^{2}}{n}}$$

#### 2.3.2. Exposure Assessment

We calculated the number of people exposed to PM_2.5_ levels exceeding the WHO target values [36]. WHO sets the air quality guideline (AQG) and interim targets of annual PM_2.5_ concentration, as shown in Table 1. The PM_2.5_ concentrations and population grids were overlaid to calculate the portion of the population exposed to PM_2.5_ pollution at different levels on a national scale. Furthermore, twofold Interim Target-1 (2IT-1, 70 μg/m^3^) and fourfold IT-2 (4IT-2, 100 μg/m^3^) were introduced as additional thresholds to quantify PM_2.5_ pollution levels because the Chinese PM_2.5_ concentration has a range of more than 100 μg/m^3^, which is far beyond the AQG of WHO.

#### 2.3.3. Health Risks and Economic Benefits Evaluation

The BenMAP Community Edition (BenMAP-CE) program was used to estimate the human health impacts. BenMAP-CE is an open source, Windows-based computer program created by the US Environmental Protection Agency (EPA) that estimates the health benefits from improvements in air quality. The estimated results can provide scientific support for air quality management and decision making. BenMAP-CE applies Equation (6) to calculate health impacts:

(6) $$\Delta Y=Y_{0}\left(1-e^{-\beta \Delta \mathrm{PM}}\right)\times \mathrm{Pop}$$

where ΔY is the estimated health impact attributed to air pollution, $Y_{0}$ is the baseline incidence, and β is the parameter (empirical value) associated with the type of pollutant used to calculate the health impact. ΔPM refers to air quality change, and Pop is the exposed population number.

The economic value of avoided premature mortality is generally calculated using the value of statistical life (VSL). The VSL is the monetary value that a group of people are willing to pay to slightly reduce the risk of premature death in the population. Moreover, the BenMAP-CE database includes several functions for VSL and valuation functions for other health effects, and we selected the function that utilizes the EPA’s mean VSL values.

## 3. Results

### 3.1. Spatioemporal Patterns and Variations in PM_2.5_

**Figure 2:**
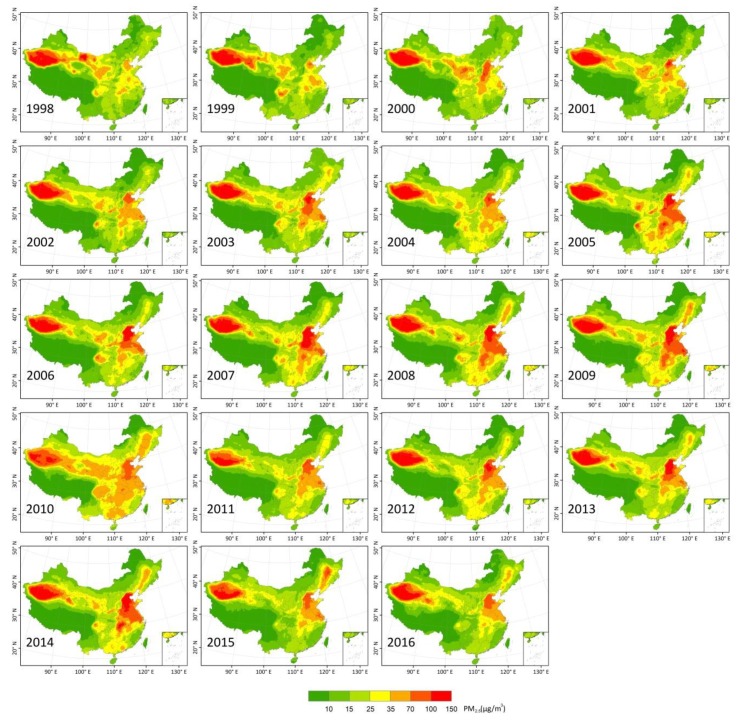
Spatial distribution of annual mean PM_2.5_ concentrations in China from 1998 to 2016.

Figure 2 shows annual mean satellite-derived PM_2.5_ concentrations over China from 1998 to 2016. In general, the annual mean PM_2.5_ concentrations showed obvious spatial and temporal variation during study periods. Higher PM_2.5_ concentrations are visible in eastern China and western Tarim Basin, which covers northwest China with its largest, driest, and highest desert. Lower concentrations are distributed in forested regions of the northeast and southwest.

The temporal variations reflect three stages during the study period. In phase 1 (~1998 to 2008), there was a rapid increase in high PM_2.5_ concentrations, with peaks during 2008 over the heavily polluted region of Beijing-Tianjin-Hebei (BTH). Phase 2 followed with a fluctuation change in PM_2.5_ concentration from 2009 to 2013. After that, the PM_2.5_ concentration had a decreasing trend in the third phase since 2014 due to the Air Pollution Prevention and Control Action Plan (APPCAP) implemented by the Chinese government.

**Figure 3:**
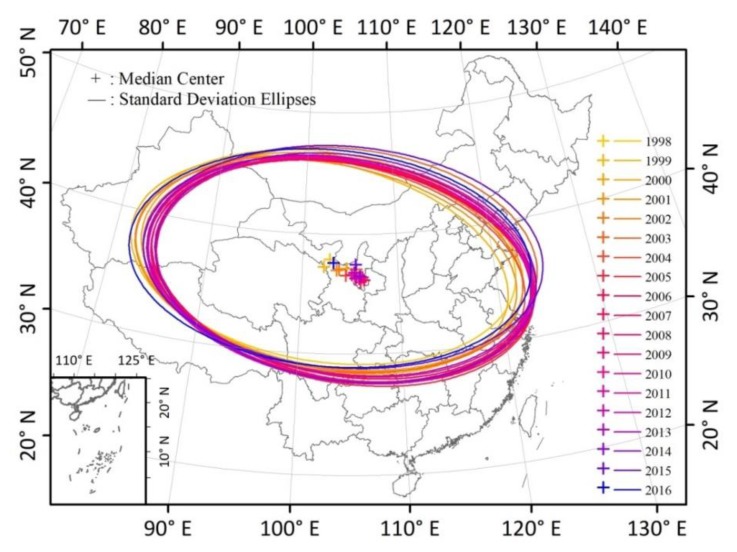
Spatial changes in the median center and standard deviational ellipses (SDEs) of PM_2.5_ concentrations in China from 1998 to 2016.

The overall spatial pattern changes in PM_2.5_ concentration across China from 1998 to 2016 were determined through standard deviational ellipse analysis (Figure 3). The median centers shifted from north central China toward the southeast and then returned during the examined time period. Longitude fluctuated from 102.91° to 106.49° and then to 103.31°, and latitude fluctuated from 37.88° to 36.16° and then to 37.60° (Table 2). Rapid increases in PM_2.5_ in the BTH metropolitan area led to the movement of the median center to the southeast from 1998 to 2013. After that, improved PM_2.5_ in eastern China contributed to the center moving northwest.

The major and minor axes of the ellipses indicate the directions and ranges of the data distribution. In this study, the major axis of the ellipse increased from 1746.5 km in 1998 to 1837.9 km in 2016, and the minor axis slightly increased from 955.3 km in1998 to 988.9 km in 2016. The increase of the two axes illustrates a spatial diffusion tendency and even spatial changes in PM_2.5_ concentration during the study period. The increase of the major axis and lengthening of the minor axis show that the range of influence of PM_2.5_ concentration increased in both the south-north and east-west directions. The azimuth of SDE can reflect the change tendency in spatial direction. During the study period, the azimuth changed from 98.7° to 102.7° and then back to 95.3°, which means that the major axis rotated clockwise and then anticlockwise. This indicates that the distribution and orientation of growing PM_2.5_ concentration changes in China were influenced from southeast to northeast during 1998 to 2016, which is partly consistent with the change of time series.

### 3.2. PM_2.5_ Exposure Assessment

**Figure 4:**
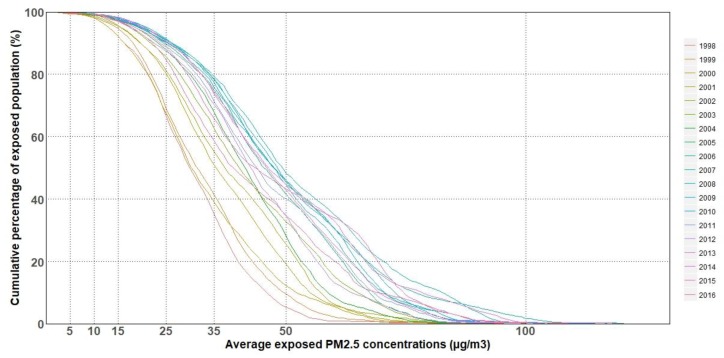
Cumulative distribution of proportion of population exposed to annual mean PM_2.5_ for 1998–2016.

Figure 4 shows the cumulative distribution of the proportion of the population exposed to annual mean PM_2.5_ concentrations, and annual population distribution averaged from 1998 to 2016 based on population raster data statistics (Figure 4). Generally, the proportion of the population exposed to the same PM_2.5_ concentrations increased at first, then there was a downward trend. Horizontally, the background PM_2.5_ levels of the same proportion of the exposed population increased. Specifically, the proportion of the total population exposed to PM_2.5_ > 100 µg/m^3^ increased from 0 in 1998 to 2.1% in 2006 and to 0.8% in 2016, whereas the proportion exposed to >70 µg/m^3^ increased from 0.7% in 1998 to 21.2% in 2007 and to 10.4% in 2016. Likewise, the proportion of the population exposed to PM_2.5_ concentrations greater than the WHO IT-1 (35 µg/m^3^) increased from 37.2% in 1998 to 78.5% in 2007 and to 57.8% in 2016, while the proportion exposed to PM_2.5_ concentrations greater than the WHO IT-2 (25 µg/m^3^) increased from 66.5% in 1998 to 91% in 2008 and to 83.2% in 2016, and the proportion exposed to PM_2.5_ concentrations greater than the WHO IT-3 (15 µg/m^3^) increased from 91.1% in 1998 to 96.5% in 2016. In addition, the proportion of the population exposed to PM_2.5_ concentrations greater than the WHO AQG value of 10 µg/m^3^ increased from 97.6% in 1998 to 99% in 2016.

### 3.3. Health Impact Due to PM_2.5_ Pollution

**Figure 5:**
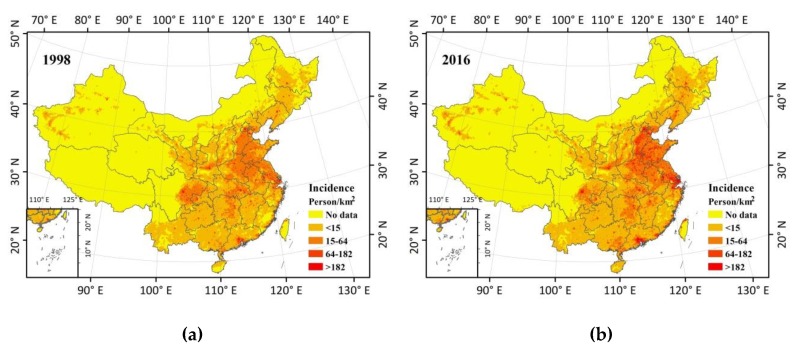
Health benefits from a hypothetical rollback of PM_2.5_ to the 10 µg/m^3^ AQG in (**a**) 1998 and (**b**) 2016.

The higher the baseline health risks of a particular location, the more premature deaths can be avoided. We calculated health incidence in the scenario of concentration of PM_2.5_ reduced to the WHO AQG value (10 µg/m^3^) based on BenMAP-CE. Because of the vast numbers of people congregating in the areas, PM_2.5_ concentrations are greater than the WHO AQG value of 10 µg/m^3^ in China (Figure 1 and Figure 4). In the result (Figure 5), the health incidences (number of incidents averted per year per 1 km × 1 km grid cell if PM_2.5_ is rolled back to 10 µg/m^3^) were categorized into low, medium, high, and extremely high incidence, corresponding to <15, 15–64, 64–182, and >182 persons/km^2^, respectively. No data means there was no population data in that area.

Figure 5 shows the spatial distributions of health incidence when PM_2.5_ is rolled back to 10 µg/m^3^ in 1998 and 2016 in China. It shows that if we reduce the PM_2.5_ concentrations in 1998 and 2016 to 10 µg/m^3^, the number of avoided premature deaths ranges from single digits to hundreds. It presents an obvious expansion in high (64–182 persons/km^2^) and extremely high (>182 persons/km^2^) incidence areas from 1998 to 2016. Moreover, the average number of avoided premature deaths increased from 8.4 persons/km^2^ in 1998 to 12.1 persons/km^2^ in 2016 if PM_2.5_ concentrations were reduced to 10 µg/m^3^. It is easy to see that the spatial distributions of incidence were consistent with the distributions of the population. So although the concentrations of PM_2.5_ were high in Tarim Basin in the northwest of China, the health incidence related to PM_2.5_ exposure was not high due to the sparse population. In 1998, the high and extremely high incidence areas were distributed in eastern Sichuan Basin and eastern China, which mainly includes the BTH, YRD, and PRD metropolitan agglomerations. The expansion of high and extremely high incidence areas also rose in China’s top three urban agglomerations by 2016.

## 4. Discussion

### 4.1. Correlation between Exposed Population and PM_2.5_

**Figure 6:**
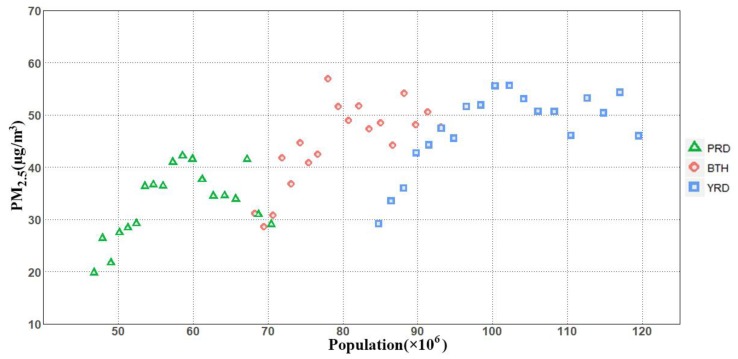
Scatter plot of population and PM_2.5_ concentrations of Beijing-Tianjin-Hebei (BTH), Yangtze River Delta (YRD), and Pearl River Delta (PRD) from 1998 to 2016.

As previously mentioned, Figure 4 gives the cumulative distribution of the proportion of the population exposed to annual mean PM_2.5_ concentrations on a national scale. However, the correlation between exposed population and PM_2.5_ is complex. In order to reveal the correlation, we chose China’s top three urban agglomerations, BTH, YRD, and PRD, which have both large populations and high PM_2.5_ concentrations, to do further statistical analysis (Figure 6).

The increasing population and PM_2.5_ concentrations in BTH, YRD, and PRD from 1998 to 2016 are shown in Figure 6. In general, both increased in 2016 compared with 1998. The PM_2.5_ concentrations of the three regions have the same change trend with the change of population; that is, increasing first and then fluctuating down in the time series. However, due to differences in population base and PM_2.5_ concentration, the range of variation varies from place to place. Specifically, the PM_2.5_ concentration in PRD is relatively lower than in YRD and BTH. YRD had the highest population, followed by BTH and PRD. Similarly, YRD had higher increased population than BTH and PRD.

Due to regional differences, the influx of the Chinese population into the eastern coastal areas has increased the level of poverty and regional imbalances. This was also the main reason why the PM_2.5_ concentrations in BTH, YRD, and PRD had the largest increases, with the population increasing at the level of millions between 1998 and 2016. However, we were delighted to find that at the end of the study period, YRD, PRD, and BTH had declines in PM_2.5_ concentrations monitored and controlled by government regulations in spite of the increasing population.

### 4.2. Estimating Health Benefits on Air Pollution Prevention and Control Action Plan

**Figure 7:**
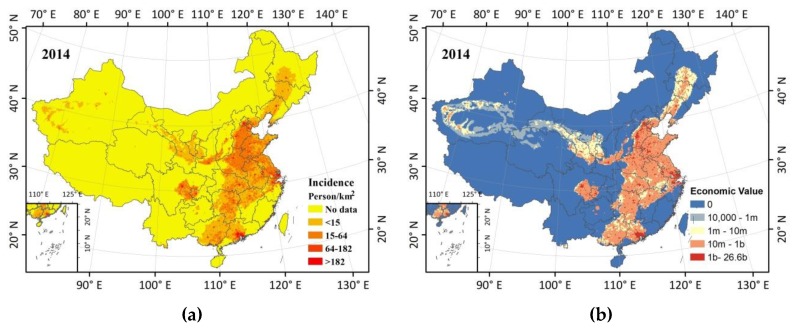
(**a**) Health incidence and (**b**) economic value of reductions in mortality (m, million; b, billion) when PM_2.5_ is rolled back to WHO’s IT-1 in 2014.

Since the end of 2013, the Chinese government has been implementing a targeted air pollution control action plan (APPCAP) that has improved air pollution and reduced PM_2.5_ concentrations. A strategic target of APPCAP was that from 2015, the Pearl River Delta, the Yangtze River Delta, and the Beijing-Tianjin-Hebei Region would meet an annual PM_2.5_ standard of 35 µg/m^3^ in three sequential five-year plans and achieve the WHO IT-1 (35 µg/m^3^) standard nationwide by 2030. We estimate that potential PM_2.5_-related premature deaths and the economic value of reductions in mortality would be avoided by meeting an annual PM_2.5_ standard of 35 µg/m^3^ using BenMap-CE (Figure 7).

High incidence and economic value of reductions in mortality were still distributed in eastern China and Sichuan Basin (Figure 7), which have similar characteristics with PM_2.5_ concentrations and population in space distribution. Coincidentally, economic value and incidence have similar distribution characteristics when the two illustrations in Figure 7 are compared. In particular, the estimated monetary value of avoided cases of all-cause mortality range from 10,000 up to 26.6 billion CNY (at a 10 km grid level), which accounts for 1.2% of 2014 gross domestic product (GDP) [37] of Beijing assuming APPCAP was completed.

Although improvements to these estimates are needed to reduce uncertainties, exposure to PM_2.5_ has already threatened human health, which cannot be ignored, due to China’s rapid urbanization. At the same time as controlling air pollution emissions, the government should take effective measures to limit the scale of more densely populated megacities and turn to developing less dense small- and medium-sized cities. In general, this also reflects that the regional inequality in China is an urgent problem to solve.

## 5. Conclusions

In this work, we analyzed the spatial patterns and temporal trends of PM_2.5_ concentrations in China based on long-term satellite-derived PM_2.5_ datasets. We also examined long-term exposure and changes in annual concentrations from 1998 through 2016. Moreover, the health impact due to PM_2.5_ pollution was estimated. We got the following conclusions.

(1) Throughout the study period, the concentrations of PM_2.5_ increased nonlinearly in most areas of China from 1998 to 2016. The center of the average PM_2.5_ concentration on the national scale shifted from north central to southeast, then returned to north central during the study years.

(2) The proportion of the population exposed to high PM_2.5_ concentrations increased annually at first, and the condition has improved since APPCAP was carried out at the end of 2013. In addition, the growth in the proportion of the population exposed between the WHO IT-2 (25 µg/m^3^) and 2IT-1 (70 µg/m^3^) was significantly faster than with exposure to other PM_2.5_ concentrations.

(3) Health risk in China increased from 1998 to 2016, and expanded in high and extremely high risk areas. Air pollution control plans are urgently needed in China’s top three urban agglomerations, which share the health risk from high to extremely high levels.

(4) Since the implementation of APPCAP, both the proportion of the population exposed to high PM_2.5_ concentrations and the annual average concentration have decreased. If a long-term approach is taken, the economic value of reductions in mortality will range up to 26.6 billion CNY (2014 CNY) per 100 square kilometers.

## Figures and Tables

**Table 1:** World Health Organization (WHO) air quality guidelines and interim targets for particulate matter with an aerodynamic diameter of less than 2.5 micrometers (PM_2.5_): annual mean concentrations.

Level	PM_2.5_ (µg/m^3^)	Basis for Selected Level
Interim Target-1 (IT-1)	35	These levels are associated with about a 15% higher long-term mortality risk relative to the AQG level.
Interim Target-2 (IT-2)	25	In addition to other health benefits, these levels lower the risk of premature mortality by approximately 6% (2%–11%) relative to the IT-1 level.
Interim Target-3 (IT-3)	15	In addition to other health benefits, these levels reduce mortality risk by approximately 6% (2%–11%) relative to the IT-2 level.
Air quality guideline (AQG)	10	These are the lowest levels at which total, cardiopulmonary, and lung cancer mortality have been shown to increase with more than 95% confidence in response to long-term exposure to PM_2.5_.

**Table 2:** Standard deviational ellipse features on median center, major axis, minor axis, and azimuth in China from 1998–2016.

Year	Median Center (°)		Major Axis (km)	Minor Axis (km)	Azimuth (°)
	Longitude	Latitude			
1998	102.91	37.88	1746.5	955.3	98.7
1999	102.31	37.25	1750.1	941.4	102.7
2000	104.66	37.14	1758.5	962.9	99.6
2001	103.64	36.99	1763.0	928.7	100.9
2002	103.90	37.04	1784.5	951.9	99.9
2003	105.70	37.07	1785.0	1019.4	95.5
2004	104.58	36.54	1764.6	979.2	101.4
2005	106.14	36.02	1766.3	1005.3	100.9
2006	105.97	36.43	1745.6	971.1	100.1
2007	106.49	36.16	1747.5	979.1	100.9
2008	105.70	36.36	1775.8	1016.9	99.4
2009	106.30	36.40	1742.6	1010.5	98.7
2010	105.21	36.61	1785.9	976.7	99.3
2011	105.97	36.27	1756.6	996.7	99.2
2012	105.59	36.31	1768.6	1008.7	100.1
2013	105.46	36.79	1763.2	983.2	98.6
2014	106.10	36.48	1748.0	1028.9	98.2
2015	105.63	37.48	1824.2	1011.3	94.1
2016	103.31	37.60	1837.9	988.9	95.3
